# Sialorrhea: Anatomy, Pathophysiology and Treatment with Emphasis on the Role of Botulinum Toxins

**DOI:** 10.3390/toxins5051010

**Published:** 2013-05-21

**Authors:** Amanda Amrita Lakraj, Narges Moghimi, Bahman Jabbari

**Affiliations:** 1 Department of Neurology, Yale School of Medicine, 15 York Street LLCI-920 New Haven, CT 06520, USA; E-Mail: amanda.lakraj@yale.edu; 2 Department of Neurology, Case Western Reserve University; Cleveland, OH 44106, USA; E-Mail: narges_moghimi@yahoo.com

**Keywords:** botulinum neurotoxins, botox, drooling, pytialis, double-blind, therapy, topical agents, oral agents

## Abstract

Sialorrhea or excessive drooling is a major issue in children with cerebral palsy and adults with neurodegenerative disorders. In this review, we describe the clinical features, anatomy and physiology of sialorrhea, as well as a review of the world literature on medical treatment using Yale University’s search engine; including but not limited to Medline and Erasmus. Level of drug efficacy is defined according to the guidelines of American Academy of Neurology. Current medical management is unsatisfactory. Topical agents (scopolamine and tropicamide) and oral agents (glyccopyrolate) combined render a level B evidence (probably effective); however, this treatment is associated with troublesome side effects. Double-blind and placebo-controlled studies of botulinum toxin (BoNT) provide a level A evidence for type B (two class I studies; effective and established) and both overall and individual B level of evidence for OnabotulinumtoxinA (A/Ona) and AbobotulinumtoxinA (A/Abo); these are probably effective. For IncobotulinumtoxinA (A/Inco), the level of evidence is U (insufficient) due to lack of blinded studies. Side effects are uncommon; transient and comparable between the two types of toxin. A clinical note at the end of this review comments on fine clinical points. Administration of BoNTs into salivary glands is currently the most effective way of treating sialorrhea.

## 1. Introduction—Definition and Incidence

Sialorrhea, also known as drooling or ptyalis, is a debilitating symptom which occurs when there is excess saliva in the mouth beyond the lip margin [1]. Drooling is common in normally developed babies but subsides between the ages 15 to 36 months with establishment of salivary continence [1]. It is considered abnormal after age 4 [2]. Pathologic sialorrhea can be an isolated phenomenon due to hypersalivation or occur in conjunction with several neurologic disorders such as amyotrophic lateral sclerosis (ALS), cerebral palsy (CP), Parkinson’s disease (PD), or as a side effect of medications. In children, the most common cause of sialorrhea is CP, which persists in 10%–38% of these individuals [3]. In adults, PD is the most common cause [4] with 70%–80% of PD patients demonstrating sialorrhea [5]. In 30%–80% of schizophrenic patients, hypersalivation when taking clozapine is manifested [6]. Regardless of the cause, drooling is problematic, leading to clinical and functional complications such as impairment in social functioning (embarrassment and isolation), aspiration, skin breakdown, bad odor, and infection [7]. 

The major salivary glands include parotid, submandibular, and sublingual glands; the largest being the parotid gland. These glands secrete saliva which has a major role in lubrication, digestion, immunity and maintenance of homeostasis in the human body [8]. The parotid gland is located in the preauricular region, along the posterior surface of the mandible and is divided by the facial nerve into a superficial lobe and a deep lobe. The submandibular gland is the second largest major salivary gland, is located in the submandibular triangle, and lies posterolateral to the mylohyoid muscle. The sublingual gland is the smallest of the three and lies in the anterior floor of the mouth [9]. Saliva is produced in high volumes relative to the mass of each gland (parotid produces the most because it is the largest) and is almost completely controlled extrinsically by the autonomic nervous system, both sympathetic and parasympathetic divisions [8]. 

In the unstimulated state, 70% of saliva is secreted by submandibular and sublingual glands. Conversely, in the stimulated state the parotids glands provide most of the saliva. The flow of saliva is five times greater in the stimulated state than in the resting state [7]. An example of an exogenous source causing stimulation is chewing. 

Sialorrhea can be either due to increased production of saliva (idiopathic or drug-induced) or related to failure of mechanisms that clear and remove saliva from the oral cavity. Disturbance in the coordination of orofacial and palate—lingual musculature is one mechanism that can lead to pooling of saliva in anterior portion of mouth. Ultimately, muscle incoordination inhibits the initiation of the swallow reflex, thereby further disrupting the path of saliva from the mouth to the oropharynx [10]. Salivary secretion is regulated via a reflex arch which has various influences. The afferent branch consists of chemoreceptors in taste buds and mechanoreceptors in the periodontal ligament. Afferent innervations of cranial nerves V, VII, IX and X also play a role by carrying impulses to salivary nuclei in the medulla oblongata [11]. Efferent influences are mainly parasympathetic via cranial nerve VII which control the submandibular, sublingual, and other minor glands, and CN IX which influences the parotid gland [11]. 

Sialorrhea occurring with neurologic illnesses is usually due to impaired swallowing as a result of impaired neuromuscular function. Neuromucular activity of swallowing involves efficient coordination of several structures including the oral cavity, pharynx, larynx, and esophagus [7]. These structures coordinate to form three phases; an oral phase which is under voluntary control, followed by the pharyngeal and esophageal phases which are under involuntary control [12]. Spontaneous swallowing is necessary for drool control [7]. In children with neurologic disorders, drooling appears to be an effect of inefficient tongue and/or bulbar control, rather than increased salivary secretion [13].

## 2. Objective

The objective of this paper is to provide a comprehensive review of sialorrhea and its various neurologic presentations, thus providing information on anatomy, physiology, and current treatment methods with an emphasis on the role of botulinum neurotoxins (BoNTs). The evidence for treatment efficacy is presented according to the guidelines of the American Academy of Neurology [14] (Table 1). 

**Table 1:** AAN classification of evidence [14,15].

Class	Criteria	Level of Evidence	Recommendation
I	Prospective, randomized, controlled, outcome masked, representative population with criteria A–E *	A: Two or more Class I studies	Established as effective, ineffective, or harmful
II	Prospective, matched cohort, representative population, masked outcome and meets A–E * OR RCT with one criteria in A–E * lacking	B: At least one Class I or two Class II	Probably effective, ineffective, or harmful and recommended
III	Controlled trial **, representative population, outcome independent of patient treatment	C: At least one Class II	Possibly effective, ineffective or harmful, may be used at discretion of clinician
IV	Uncontrolled study, case series, case report or expert opinion.	U	Data inadequate or conflicting

* A = Primary outcome(s) clearly defined, B = exclusion/inclusion criteria clearly defined, C = Adequate accounting for drop-outs and cross-over with numbers sufficiently low to have minimal potential for bias, D = relevant baseline characteristics or appropriate statistical adjustment for differences, E = For non-inferiority or equivalence trials claiming to prove efficacy for one or both drugs, the following are also required: (1) The standard treatment used in the study is substantially similar to that used in previous studies establishing efficacy of the standard treatment (e.g., for a drug, the mode of administration, dose, and dosage adjustments are similar to those previously shown to be effective); (2) The inclusion and exclusion criteria for patient selection and the outcomes of patients on the standard treatment are substantially equivalent to those of previous studies establishing efficacy of the standard treatment; and (3) The interpretation of the results of the study is based on an observed-cases analysis; ** Including well-defined natural history controls or patients serving as their own controls.

## 3. Method

Information for this paper was collected by searching the Yale Medical Library Database, which utilizes a wide range of scholarly search engines including, but not limited to, Pubmed, Erasmus, Ovid, EBSCO and Cochrane databases. Literature was searched from the time line 1960 to present, including literature ahead of print and articles not in English. Terms used for search included “saliva”, “drooling”, “sialorrhea”, “hypersalivation”, “ptyalis”, “double-blind”, “therapy”, “treatment”, “botulinum toxin”, and “botulinum neurotoxin” in various combinations.

## 4. Treatments

Sialorrhea is known to be difficult to treat. Management can be conservative or more invasive. Conservative treatments include changes in diet or habits, oral-motor exercises, intra-oral devices such as palatal training devices, and medical treatments such as medication or botulinum toxin injections. Behavior modification has been advocated for many decades but results are inconsistent and time consuming [16]. More invasive treatments include surgery or radiation [17]. While surgical cases seem to offer more permanent results, they are invasive and are not without side effects. Radiation is now rarely applied and is typically reserved for elderly patients who are not candidates for surgery and cannot tolerate medical therapies [18].

### 4.1. Oral (Table 2)

Oral therapy for sialorrhea encompasses the use of anticholinergic agents such as glycopyrrolate, benztropine, scopalamine and tropicamide. Biperiden has also been implicated for use in the literature but was found in one study to have an adverse effect on cognition, thus limiting its use [19]. Glycopyrrolate oral solution is the first drug treatment which was approved in the United States for drooling in children with neurologic conditions [1]. Anticholinergic agents work by downregulating acetylcholine and ultimately decreasing saliva secretion through the parasympathetic autonomic nervous system [17]. Elderly patients have poor tolerance for anticholinergic agents. Glycopyrrolate specifically has a quaternary ammonium structure and thus cannot pass the blood-brain barrier in large amounts, ultimately decreasing the occurrence of central side effects [20]. It is effective and safe at 1 mg, three times a day. Intraoral tropicamide films provide short-term relief of sialorrhea. One study provided evidence that 1 mg of tropicamide resulted in significant Visual Analog Scale (VAS) score decrease and reduction in saliva volume in non-demented PD patients [21]. Antireflux medication has also been suggested for use in drooling [22]; however, there are no double-blind studies in the literature to offer evidence for this recommendation as per our search. 

#### Level of Evidence

Six double-blind studies investigated the efficacy of oral agents in sialorrhea (Table 2). The majority focused on oral anticholinergics (glycopyrrolate or benztropine). One investigated the use of intraoral tropicamide films, and the other studied the efficacy of sublingual ipratroprium bromide. These six studies are comprised of three class II and three class III studies, yielding an overall B level of evidence (probably effective).

**Table 2:** Treatment of sialorrhea with pharmacological oral and topical agents.

Author and year	Agent	Associated illness	*N*	Study design	Class	Outcome measured	Findings	Side effects
Camp-Bruno *et al*. 1989 [23]	BZ (mean dose 3.8 mg)	DD	20	DB, PBOC, CO	III	Efficacy and incidence of side-effects at 1-week, baseline, 2-week PBO and 2-week BZ conditions	A significant decrease in drooling during the BZ condition relative to PBO was demonstrated and conservative response rates ranged up to 65%–70%	Minor problems (dry mouth) eliminated by small dose adjustments. More serious cholinergic side-effects which resolved in 24–48 h required discontinuations of the drug in three patients.
Mier *et al*. 2000 [24]	GLYC	DD	39	DB, PBOC, CO, Dose-ranging	III	Parent and investigator evaluation of change in sialorrhea and adverse effects	GLYC in doses of 0.10 mg/kg per dose is effective at controlling sialorrhea	Even at low doses, 20% of children may exhibit adverse effects severe enough to require drug discontinuation.
Thomsen *et al*. 2007 [25]	Sublingual Ipratropium Bromide Spray (1–2 sprays up to 4 times a day)	PD	17	Randomized, DB, PBOC, CO	II	Objective measure of weight of saliva production	Ipratopium bromide spray had no significant effect on weight of saliva produced.	No significant adverse side effects
Mato *et al*. 2010 [26]	Topical Scopolamine	Handicapped *	30	prospective, randomized, DB, PBOC, CO	II	Severity of drooling was quantified using a modified Thomas-Stonell and Greenberg visual scale simplified into three grades: 1 = dry; 2 = mild/moderate; 3 = severe/fulsome. The frequency of drooling was estimated using the number of bibs used each day.	Significant drooling reduction (*p* < 0.005) in the scopolamine group in the 1 and 2 week controls (69% and 80% respectively ≤ grade 3). The mean number of bibs/day decreased during the scopolamine phase from 6/day at baseline to 3/day at the 2 week control.	4 patients (13.3%) dropped out because of scopolamine side effects and minor adverse reactions were observed in three other patients
Arbrouw *et al*. 2010 [20]	GLYC 1 mg 3 times daily	PD	23	4-week, randomized, DB, PBOC, CO	II	Sialorrhea was scored on a daily basis by the patients or a caregiver with a sialorrhea scoring scale ranging from 1 (no sialorrhea) to 9 (profuse sialorrhea).	Mean sialorrhea score improved from 4.6 (1.7) with PBO to 3.8 (1.6) with GLYC (*p* = 0.011). 9 patients (39.1%) with GLYC had a clinically relevant improvement of at least 30% *vs*. 1 patient (4.3%) with PBO (*p* = 0.021).	No significant differences in adverse events between GLYC and PBO treatment.
Liang *et al*. 2010 [19]	GLYC and Biperiden	CI	13	12-week, randomized, DB, CO, fixed-dose	III	Sialorrhea and global cognitive function were assessed by using DRS and MMSE respectively	At 1 week, both drugs improved CIS compared with baseline (biperiden: *p* = 0.005; GLYC: *p* = 0.002). DRS score was lower than baseline in 4 weeks with biperiden (*p* = 0.003) and also with GLYC at 4 weeks (*p* = 0.002). MMSE scores with either drug did not differ from baseline at one week or 4 weeks (*p* = 0.437, *p* = 0.76). Patients treated with biperiden had significantly reduced MMSE scores after 1 week (*p* = 0.049).	2 adverse events were reported by the patients during both treatment phases. 1 patient complained of constipation, and the other complained of inner unrest
Lloret *et. al*. 2011 [21]	Intra-oral Tropicamide films	PD	19	DB, randomized, PBOC, two-phased, Latin-square CO study	II	VAS and saliva amount by cotton rolls.	1 mg of tropicamide resulted in significant VAS score decrease and reduction in saliva volume (27%, 33%, and 20% respective for 0.3, 1 and 3 mg) when compared to PBO	No adverse events were reported

Double Blind (DB), Placebo Controlled (PBOC), Cross over (CO), Glycopyrrolate (GLYC), Number of subjects (N), Visual Analogue Scale (VAS), Drooling Rating Scale (DRS), Mini Mental Status Exam (MMSE), Parkinson’s Disease (PD), Cerebral Palsy (CP), Developmentally Disabled (DD), Clozapine-Induced (CI). *11 cerebral palsy, 5 epilepsy, 4 autism, 3 Dpwns syndrome and 3 cases of rare disorders

### 4.2. Topical (Table 2)

One double-blind study of 30 patients handicapped by debilitating illnesses (CP, epilepsy, autism, Down syndrome and rare disorders) is described in the literature using transdermal scopolamine. Moderate or severe mental retardation was present in 80% of patients. Severity of drooling was quantified using a modified Thomas-Stonell and Greenberg visual scale and frequency of drooling was monitored by number of bibs used each day. A significant drooling reduction was noted (*p* < 0.05); however, four patients dropped out due to side effects and minor adverse events were noted in three others. It was concluded that though scopolamine can be useful to control drooling in severely disabled patients, it is not free from adverse effects, thus requiring careful patient selection [26]. 

#### Level of Evidence

This class II study justifies a C level of evidence (possibly effective) 

### 4.3. Surgical

Various surgical procedures have been suggested for treatment of drooling. These procedures all encompass an alteration in the anatomy of the salivary glands and include salivary gland excision, denervation of the salivary glands, and transposition or ligation of the salivary ducts [27]. These procedures are reserved for severe cases and of course are not without their side effects. 

## 5. Botulinum Toxin Treatment (Table 3)

The effect of Botulinum toxin (BoNT) on drooling was first noted in PD patients [28]. BoNT, a potent neurotoxin blocks the release of acetylcholine and a number of other neurotransmitters from synaptic vesicles [28]; in this case post-ganglionic parasympathetic fibers are cholinergic. It has been reported to be effective in the treatment of sialorrhea through various open label trials, retrospective studies, case studies and controlled clinical trials. Currently, three type A and one type B toxin are approved for use in US. These include OnabotulinumtoxinA (A/Ona), AbobotulinumtoxinA (A/Abo), IncobotulinumtoxinA (A/Inco), and RimabotulinumtoxinB (B/Rima). 

**Figure 1:**
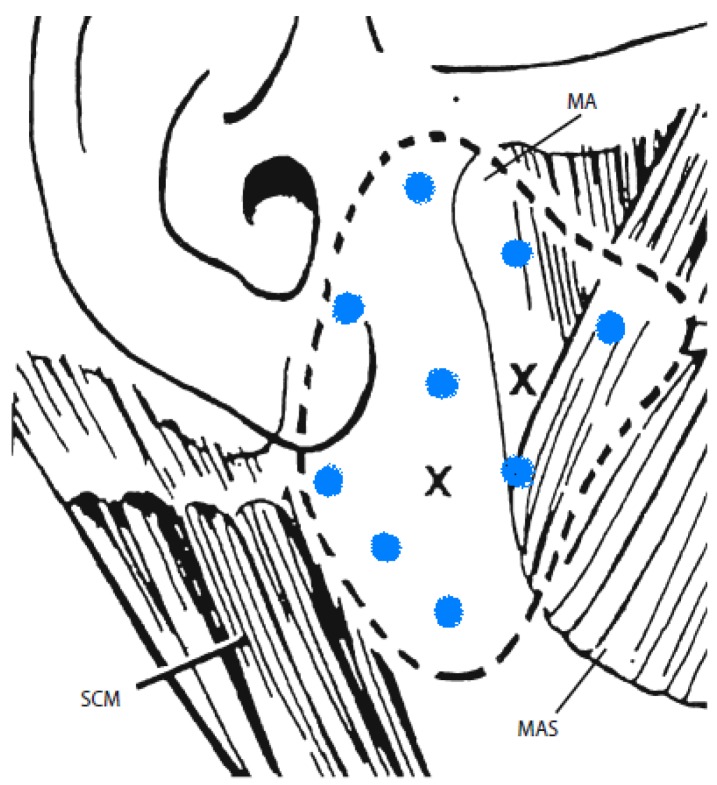
Locations for Parotid gland injections. This figure depicts the way in which Lagalla *et al*. [29] inject into the parotid gland (the black x’s). Many of thestudies have used the same approach, injecting in only 2 sites on the parotid gland. At our institution, we inject into nine different sites and have modified the figure to portray this by the blue dots. Modified with permission from Springer [29].

**Figure 2:**
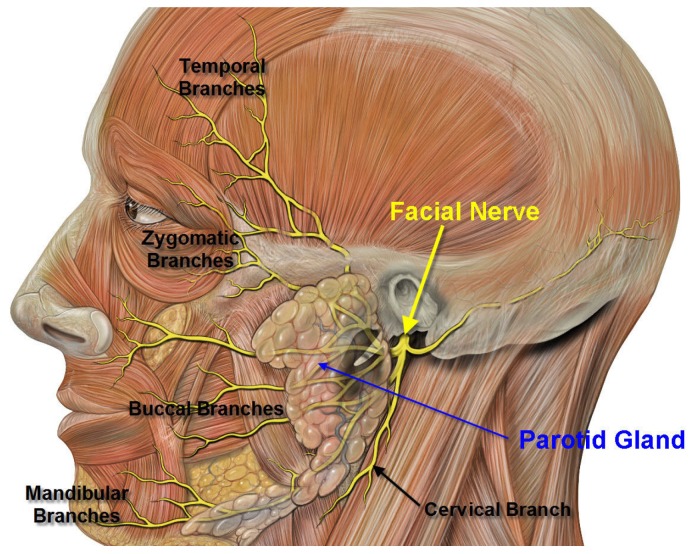
Facial Nerve location in relation to parotid gland. It is important to note the anatomical location of the facial nerve in relation to the parotid gland in order to avoid injury to this functionally important nerve during injection.

Below we provide the evidence for the use of BoNT in sialorrhea divided into three categories: BotulinumtoxinA (BoNT-A) *vs*. placebo (Table 3), BotulinumtoxinB (BoNT-B) *vs*. placebo (Table 4) and comparator studies looking at BoNT-A *vs*. BoNT-B or BoNT-A *vs*. topical (transdermal) agents (Table 5). BoNT injections are given in the parotid and submandibular glands, as they are the greatest contributors to salivary production (Figure 1 [29]). The facial nerve, which is important in facial expression, is very close to the parotid gland; caution must be taken when injecting to avoid this nerve (Figure 2 [30]). Although local anesthesia may be used to reduce the pain of injection, out of all the studies reported in this review, only two used it. In one, authors used generalized anesthesia before injection in the pediatric population [31]. In the other, local anesthesia with Emla cream was used in five of the patients [32].

**Table 3:** Double blind studies for botulinum toxin A *vs*. placebo.

Author/year	Assoc. illness	*N*	Class	Agent/dose	Glands injected	Primary outcome	Result	Side effects
Lipp *et al*. 2003 [28]	12 ALS, 12 PD, 4 MSA, 4 CBD	32	II	A/Abo 18.75, 37.5, or 75 MU	B/L PG	Weight of dental rolls every 4 weeks during a 24-week period	Significant decrease of sialorrhea during the study period measured by dental rolls when compared with PBO group *p* < 0.05 only in the 75 U group	None reported
Mancini 2003 [33]	14 PD, 6 MSA	20	II	450 U A/Abo	B/L PG and B/L SMG	Treatment efficacy and safety were assessed at baseline, 1 week and 3 months after A/Abo injections using clinical scales (DS and DF) and side effect surveillance	After treatment, the average secretion of saliva in the A/Abo group was significantly lower than in the PBO group as appraised by clinical measurements (*p* = 0.005); no treatment difference at 3 months	None
Lagalla *et al*. 2006 [34]	PD	32	II	A/Ona50 U per PG,	B/L PG	DSFS, VAS-FD and SD, UPDRS-ADL item scores for drooling and swallowing at baseline and 1 month after treatment. Saliva reduction (weight of dental rolls). GIS was also applied	Subjects treated with A/Ona experienced a reduction in both drooling frequency and familial and social disability, as well as in saliva production (*p* < 0.0001)	Mild transient swallowing difficulty in 1 pt
Lin *et al*. 2008 [16]	CP	13	III	Ona/A 2U/kg body weight	C/L PG and SMG	DSFS, saliva weight, and DQ	Significant difference in DSFS at 2, 4, 6, 8, 12 week post injection, saliva weight at 6, 12 week after injection, and DQ 2, 6, 8, 10 week after injection all significant at *p* < 0.05. Saliva weight significant to longest follow up of 22 weeks.	Not stated in text
Alrefai *et al*. 2009 [35]	CP	24	III	100 U A/Abo in the first visit and 140 U at the second visit 4 months later regardless of effect	B/L PG and SMG	DF and DS were performed at the time of injection, at 1 month, and at baseline prior to the second injection. A second set of injections of either 140 U of A/Abo or PBO was given 4 months later, and the same rating scales were used using Fisher’s Exact Test	Scores of the median frequency (*p* = 0.034) and severity (*p* = 0.026) of drooling were reduced in the treatment group.	Minor and transient increase in drooling after Injection in 2 pts *
Pei-Hsuan Wu *et al*. 2011 [36]	CP	20	I	A/Ona body weight titrated	B/L PG and SMG	Subjective drooling scales, salivary flow rate, and oral health (salivary compositions and cariogenic bacterial counts) at 1 and 3 months	Decrease in salivary flow rate was significantly higher in the A/Ona group at the 1-month (*p* = 0.037) and 3-month (*p* = 0.041) follow up compared to control	No reported adverse effects

* 2 patients experienced a mild/transient increase in drooling which the authors proposed can be explained by either improper placement of the injection or to local leakage of BoNT into surrounding muscles resulting in weakness of mouth closure.

**Table 4:** BTX-A *vs*. PBO studies: Technical information.

Author/year	Method used to locate injection site	Method of injection	Number of sites injected	Use of anesthesia/type
Lipp *et al*. 2003 [28]	Anatomic landmarks	30-gauge, 25-mm needle were used to inject each parotid gland. (one in gland mass (0.3 mL), one above masseter 0.2 mL)	2 per parotid	Not stated
Mancini 2003 [33]	Ultrasound	Through a 26-gauge syringe, 0.65 mL of solution in each parotid gland and 0.35 mL of solution in each submandibular gland.	Not clear, but assumed 1 injection per gland on both sides	Not stated, however patients complained of painful injections
Lagalla *et al*. 2006 [34]	Anatomic landmarks	27-gauge needle penetrating to adepth of 1–1.5 cm into the preauricular portion of parotid gland, behind the angle of the ascending mandibular ramus, and then into the inferoposterior portion of the gland, lying just before the mastoid process.	3 per parotid	Not states
Lin *et al*. 2008 [16]	Ultrasound	Not stated in paper	Not stated in paper	Not stated
Alrefai *et al*. 2009 [35]	Anatomic landmarks	Each side was injected with a 10 mm (30 G) needle into the paotid gland with 50 U.	2 per parotid	Not used
Pei-Hsuan Wu *et al*. 2011 [36]	Ultrasound	30-gauge needle to the bilateral parotid and submandibular glands with concentration of 10 U/0.1 mL.	4 (1 injection in both parotids and submandibular glands)	Not stated

**Table 5:** Comparator studies.

Author/year	Assoc. illness	*N*	Class	Agent/dose	Glands injected	Primary outcome	Result	Side effects
Jongerius *et al.* 2004 [32]	CP	45	III	A/Ona by weight *vs.* transdermal scopolamine	B/L SMG	DQ, Teacher Drooling Scale (TDS) and VAS	Drooling decreased with both scopolamine and A/Ona injection; however greatest reductions were achieved 2 to 8 weeks after A/Ona injection. 61.5% of patients responded to BoNT injections. Statistical significance for DQ was stated at (*p* < 0.05). DQ had a response rate of 53% for scopolamine and 48.7% for A/Ona	71% had moderate to severe side effects for scopolamine. Only minimal and incidental side effects were reported for BoNT.
Wilken *et al.* 2008 [31]	CP or NDD	30	III	100 /kg of B/Rima or 80 MU of A/Ona	B/L PG and SMG	Parent questionnaire and TDS	Four weeks after the first injection 29 patients responded with a reduction of TDS score to 1 or 2 rated in the parent’ s questionnaire.	Intermittent problems with swallowing due to viscous saliva (5), unilateral parotitis (1).
Guibaldi *et al*. 2011 [40]	ALS (15) and PD (12)	27	III	250 U A/Abo or 2,500 U B/Rima	B/L PG and SMG	Magnitude of change in saliva production determined by weighing five cotton rolls after retaining for 5 minutes in the mouth.	B/Rima showed improvement in subjective and objective measured with a shorter latency for improvement onset when compared to A/Abo (*p* = 0.002). Mean benefit of duration was 75 days for A/Abo and 93 days for B/Rima.	Change in saliva thickness

CP—Cerebral Palsy, NDD—Neurodegenerative Disorder, ALS—Amyotrophic Lateral Sclerosis, PD—Parkinson’s Disease, DQ—Drooling quotient, TDS—Teacher Drooling Score, BoNT—Botulinum Neurotoxin, A/Ona—OnabotulinumtoxinA, B/Rima—RimabotulinumtoxinB, VAS—Visual Analog Scale, A/Abo—ABobotulinumtoxinA, B/L—Bilateral, PG—Parotid Gland, SMG—Submandibular gland.

## 6. Commentary on Studies

We have determined the level of evidence for BoNT efficacy based on reviewed studies (Table 3, Table 4, Table 6). The data on children is limited. In adults, the level of evidence for BoNT-A (A/Ona, A/Abo, and A/Inco) overall, when combining both placebo-controlled and comparator studies, is Level B. Both A/Ona and A/Abo have level B evidence individually, as well. A/Ona evidence is based on five studies (1 class I, 1 class II, and 3 class III). A/Abo evidence is based on four studies: three class II and 1 class III study. For A/Inco the level is U (insufficient evidence) due to the lack of blinded studies. Level of evidence for botulinum toxin B (B/Rima) is A (established efficacy), based on two class I, three class II and one class III. 

As implied from this data, there is now strong evidence that both A and B types of BoNTs are effective in treatment of sialorrhea and both have a low profile of side effects. Despite the strong level of evidence collectively, the cited studies are not without limitations. One such limitation is that of outcome measures. There is significant heterogeneity of outcome measures among the studies. In some studies outcome measures were poorly defined. Some studies measured only qualitative clinical scales (drooling and activity of daily living) [31,32,33,35,37,38,39], others measured quantitative data such as saliva flow through weight of dental rolls [28,40] and yet others measured both [16,29,34,36]. Outcome measures were also evaluated at different time points (for most studies at four weeks). 

Another limitation is variability of injection technique. A recent review paper stated that factors affecting diffusion and spread of BoNT include dose, concentration and volume of injectate, number of injections, injection site, rate of injection, gauge of needle, and distance of needle tip from the neuromuscular junction [42]. The tables with technical information (Table 4, Table 7, Table 8) depict this variability regarding doses, sites of injection and use of ultrasound. The optimal dose and dilution concentration (1:1, 2:1, 4:1) still deserve further investigation. 

In regards to duration of effect, one study suggested a length of duration up to six months [40], whereas most others reported three months.

For location of injection, the studies presented focus on injections into the salivary glands (parotid and submandibular). Only one study discussed experience with direct injection into sublingual glands under the tongue with BoNT-B which lead to occurrence of dysphagia in two of four patients without any improvement in sialorrhea, thus discouraging this approach [37].

A lack of correlation between reduction in salivary secretion and improvement in drooling has been observed in some studies and is probably related to the fact that factors influencing the severity of drooling and reduction of saliva secretion are variable [51]. 

Side effects in general were few and mild. Among them, transient dysphagia was the most worrisome, resolving in two weeks and affecting a small number of patients. 

One comparator study suggested better efficacy for type B toxin. Another indicated more side effects with type B. These need to be further investigated with weighing the efficacy *vs*. side effects.

**Table 6:** Double-blind studies for botulinum toxin B *vs.* placebo.

Author/year	Assoc. illness	N	Class	Agent/dose	Glands injected	Primary outcome	Result	Side effects
Ondo 2004 [37]	PD	16	II	B/Rima 2500 U (1000 U in each PG)	PG and SMG	UPDRS, Drooling Rating Scale, DSFS, VAS, GIS at baseline and one month, drooling and dysphagia questionnaires	Improvement on the VAS (*p* <0.001), GIC (*p* < 0.005), Drooling Rating Scale (*p* < 0.05), and DFSS (*p* < 0.001). There was no change in UPDRS, head posture, or Dysphagia Scale	Dry mouth (3), worsened gait (2), diarrhea (1), neck pain (1) in B/Rima group
Jackson *et al*. 2009 [38]	ALS	20	I	B/Rima 2500 U (500 U in PG, 750 U in SMG)	B/L PG and SMG	GIC by the subject 8 weeks after the injection.	GIC of 82% at 2 weeks compared to 38% of those who received PBO (*p* < 0.05). This significant effect was sustained at 4 weeks. At 12 weeks, 50% of patients who received B/Rima continued to report improvement compared to 14% of those who received PBO.	No adverse side effects
Lagalla 2009 [29]	PD	36	II	4000 U B/Rima	PG	DSFS, VAS-FD and VAS-SD, UPDRS-ADL scores for drooling and swallowing at baseline and one month after treatment. Objective saliva reduction (saliva production over five minutes by weighing dental rolls), GIS	One month after injections, B/Rima group showed improvement in almost all subjective outcomes. Two-way analysis of variance gave a significant time × treatment effect, *F*-value being 52.5 (*p* < 0.0001) for DS-FS, 23.2 (*p* < 0.0001) for VAS-FD, 29 (*p* < 0.0001) for VAS-SD, and 28.9 (*p* < 0.0001) for UPDRS-ADL drooling item score, with benefits in B/Rima group lasting 19.2 +/− 6.3 weeks	Transient dysphagia worsening which resolved in 2 weeks in B/Rima group (3)
Chinnap-ongse *et al*. 2012 [39]	ALS and PD	54	I	B/Rima 1500, 2500 or 3500 U for PG and 250 U for the SMG	PG and SMG	Safety/tolerability, as assessed by adverse events	At 4 weeks postinjection, Drooling Frequency and Severity Scale scores significantly improved *vs*. PBO in a dose-related manner (*p* < 0.0001 and *p* < 0.0001 respectively). Unstimulated salivary flow rates significantly decreased in all active groups *vs*. PBO (*p* ≤ 0.0009).	GI-related events more frequently in the active groups (dry mouth most common)

A/Ona: Onabotulinumtoxin A (Botox); A/Inco: incobotulinumtoxinA (Xeomin); A/Abo: abobotulinumtoxinA (Dysport); B/Rima: RimabotulinumtoxinB (Myobloc), Drooling Severity (DS), Drooling Frequency (DF), Unified Parkinsonsons Disease Rating Scale (UPDRS), Activities of Daily Living (ADL), Visual Analog Scale (VAS), Visuo-analogic ratings of familial distress (VAS-FD) and social distress (VAS-SD), Global Impression Score (GIS), Drooling Frequency and Severity Scale (DFSS), Global Impression of Change (GIC), Drooling Quotient (DQ), Placebo (PBO), Bilateral (B/L), Contralateral (C/L), Parotid gland (PG), Submandibular gland (SMG), Amyotrophic Lateral Sclerosis (ALS), Parkinson Disease (PD), Multiple System Atrophy (MSA), Corticobasal Degeneration (CBD), Cerebral Palsy (CP), Number of Subjects (*N*).

**Table 7:** Double blind studies for botulinum toxin B *vs.* Placebo-technical information.

Author/year	Method used to locate injection site	Method of injection	Number of sites injected	Use of anesthesia/type
Ondo *et al*. 2004 [37]	Anatomical landmarks	All injection sites were localized with anatomic markers and injected with a 29-gauge tuberculin syringe at a depth of 0.5 inch. Two vertically placed locations just dorsal to the palpated masseter muscle (parotid gland) were injected as well as one location just anterior and medial to the genu of the mandible (submandibular gland).	2 places in both parotids and 1 site in both submandibular glands.	Not stated
Jackson *et al.* 2009 [38]	EMG—For the parotid, absence of motor unit potential was used to confirm placement. For the submandibular when insertional motor unit activity was observed (indicating myohyoid/ hyoglossus/digastric), the needle was withdrawn slightly until muscle activity was absent deep in the submandibular triangle.	Each parotid gland was injected at two sites with 0.1 cc of study medication (total of 500 U/gland) directing the needle toward the tail of the parotid, between the sternocleidomastoid muscle and the angle of the mandible. Each submandibular gland was injected at two sites with 0.15 cc of study medication (total of 750 U/gland), placing the needle percutaneously in the submandibular triangle.	Each parotid gland was injected at two sites, Each submandibular gland was injected at two sites	Not stated.
Lagalla *et al.* 2009 [29]	Anatomical landmarks	0.8 mL of drug into each pre-auricular portion of the parotid gland. The injections were performed using a 1 mL syringe with 27-gauge needle penetrating to a depth of 1–1.5 cm into two sites, behind the angle of the ascending mandibular ramus and into the infero-posterior portion of the gland, just before the mastoid process.	2 sites in each parotid	Not stated
Chinnapongse *et al.* 2012 [39]	Anatomical landmarks	Not described	Not described	Not stated

**Table 8:** Technical information for Comparator Studies.

Author/year	Method used to locate injection site	Method of injection	Number of sites injected
Jongerius *et al*. 2004 [32]	Ultrasound	Injected bilaterally in the submandibular glands using a 25-G needle * after general anesthesia	3 per submandibular gland
Wilken *et al*. 2008 [31]	Ultrasound	Injected into parotid (one in front of the isthmus and the other one below) and submandibular using 27G needle *after local anesthesia with 20% Emla in 5 pts.	3 site per side: 2 in the parotid and one in the submandibular gland
Guibaldi *et al*. 2011 [40]	Ultrasound	Not clearly stated	parotid gland (two sites per gland) and each submandibular gland (one site per gland)

**Table 9:** Summary of reviews discussing use of BoNT for sialorrhea.

Author & year	Focus of review	Conclusions	Comments
Reddihough *et al.* 2010 [41]	Adult and children with different etiologies	Study suggests established evidence (Level A) for both BoNT-A and BoNT-B	Conclusions based on randomized controlled trials, systematic reviews, AAN criteria but for some studies class rating does not accord with AAN rating (example Alrefai et al. is rated class I; rated class III by AAN search members).
Lim *et al.* 2010 [42]	Discusses the use of botulinum toxin in neurologic practice	Suggests level B evidence for BoNTs in sialorrhea (probably effective)	Conclusion based solely on two class II studies, and it is unclear which class II studies were used
Dand *et al.* 2010 [43]	Reviews the available treatments for sialorrhea (pharmacologic and non-pharmacologic)	BoNT when injected into the parotid gland may improve quality of life up to 4 months, injection into salivary ducts not recommended.	No assessment of level of evidence was cited
Habek *et al.* 2010 [44]	Botulinum toxin in the management of MS	BoNTs should be used with caution in MS since no blinded or controlled studies exist.	There are no controlled studies to date on the application in MS patients with sialorrhea.
Young *et al.* 2011 [45]	Cochrane review on individuals with MND/ ALS: BoNT, radiotherapy.	Suggested use of BoNT-B for treatment of sialorrhea in clinical practice due to better efficacy.	For BoNT efficacy only double-blind study (Jakson et al.2009) was cited and many open studies.
Seppi *et al.* 2011 [46]	An update on the efficacy of treatments for non-motor symptoms of PD based on EBM methodology using RCTs.	BoNTs A and B: established efficacy in sialorrhea. Glyccopyrolate: efficiency beyond one week is not established. Ipratropium bromide spray: insufficient data	Different used for assessment of efficacy than AAN criteria are referenced.
Squires *et al.* 2012 [47]	Adults with different neurological conditions	Pharmacologic intervention is effective but short lived. Evidence is strongest for BoNTs	Conclusions/recommendations for clinical practice brief without applying evidence based assessment criteria.
Rodwell *et al.* 2012 [48]	Systematic Review of Efficacy of BoNT in children with cerebral palsy	Data from 6 RCT suggest efficacy of BoNTs in sialorrhea. More data on adverse effects are needed.	Review is limited to children with cerebral palsy.
Intiso *et al.* 2012 [49]	Review focused of BoNT use in neurohabilitation for sialorrhea (ALS, PD and CP).	BoNTs and B are both effective in reducing drooling. Type B: more effective, shorter latency; more side effects. Duration of effect: comparable.	Does not describe levels of evidence and is not limited to highest level of evidence studies
Walshe *et al.* 2012 [50]	Interventions to treat drooling in children with CP	Unable to reach conclusion on efficacy /safety of BoNTs or pharmaceutical interventions in CP.	Looks only at the pediatric population

BoNT—Botulinum toxin, MS—Multiple Sclerosis, ALS—Amyotrophic Lateral Sclerosis, PD—Parkinson’s Disease, EBM—Evidence-Based Medicine, RCT—Randomized Controlled Trial, AAN—American Academy of Neurology, MND—Motor Neuron Disease, CP—Cerebral Palsy.

A recent meta-analysis study (2012) [22], which reviewed all randomized placebo-controlled trials encompassing 181 patients, concluded that botulinum toxin decreases the severity of drooling in patients with sialorrhea with statistical significance in both adult and pediatric populations. It was further concluded that both BoNT-A and BoNT-B produce similar effects [22]. Furthermore, BoNT-A toxin doses greater than 50 U produced stronger effects. Increased saliva thickness (3.9%), dysphagia (3.3%), xerostomia (dry mouth) (3.3%), and pneumonia (2.2%) are noted as common side effects. BoNT treatment holds many advantages over older proposed methods of therapy: limited side effect profile, convenience, low risk of aspiration and its minimally invasive nature, are just a few [22]. Limitations of using BoNT injections include expense and need for repeated sedation, which is more problematic in pediatric population. 

## 7. Conclusions

As of now, level of evidence and low rate of adverse side effects indicate that administration of BoNT-A and BoNT-B into salivary glands is the most effective way of treating sialorrhea (levels B and A evidence, respectively). Injections are simple and when executed by experienced hands, side effects are uncommon and manageable. Overall, efficacy of both toxins and their side effect profiles are comparable [49]. Special care needs to be applied when dealing with patients with ALS or those already affected by dysphagia [49]. Oral agents are probably effective (level B) but anticholinergic side effects limit their use particularly in the elderly population. Topical agents are possibly effective (one class II study level C) but effects are short lived. In the pediatric population, only three double-blind studies exist which are class III, hence rendering a U level of evidence (insufficient) [50]. 

Several reviews on sialorrhea and management have been published recently [25,41,42,43,44,46,47,48,49,50] (Table 9). The majority of these reviews have a limited scope, either focusing on a single disease category (multiple sclerosis, Parkinson’s disease, amyotrophic lateral sclerosis, cerebral palsy) or specific population (pediatric or adult). Furthermore, several of these studies lack level of evidence or, as in one case, have used a different system of efficacy assessment [46]. The report of Reddihough *et al*. is a review on the subject from a European team using the evidence criteria of the American Academy of Neurology (AAN). Due to differences in interpretation of AAN criteria, the evidence depicted in this report gives a level of evidence which is higher than levels depicted in our paper, as evidenced by the high number of class I studies in their report compared to ours. 

Our review covers the published literature on prospective and controlled studies (with placebo arm or comparator) until December 2012. It covers BoNTs, as well as oral and topical agents. The level of evidence is provided with adherence to the AAN guidelines.

## 8. Technical Note from Senior Author

Defining the correct technical approach for botulinum toxin injections in sialorrhea is still an evolving issue. Many studies which have not used ultrasound for guiding injections report good results using anatomical landmarks. Particularly, the optimum number of injections into parotid glands is still a subject of debate. Since parotid gland anatomy is variable (sometimes extending anteriorly over masseter or posteriorly and inferiorly below the angle of the jaw), we at Yale practice using a larger number of subcutaneous injections and have been doing so for the past decade. Injections are done in a grid-like pattern using three rows, with three sites injected in each row (Figure 1). The top row is at the level of tragus. This technique provided satisfaction in 90% of patients treated over the past 10 years, encompassing more than two hundred patients. Improvement is measured using patient global impression of change (PGIC). Our standard dosage is 30–40 units of A/Ona (about 0.05 cc per site) and 1250–1500 of B/Rima (about 0.03 cc per site) per parotid in adults. If parotid injection alone does not work, then a combined parotid and submandibular injection is done the next time. The submandibular injection is done at two points under the maxillary arch with a total of 30 units of A/Ona or 1000 units of B/Rima per side. We use anatomical guidelines for injections. Injections are carried out subcutaneously with a 30-gauge, half-inch needle. 
